# Management of vesicovaginal fistulas (VVFs) in women following benign gynaecologic surgery: A systematic review and meta-analysis

**DOI:** 10.1371/journal.pone.0171554

**Published:** 2017-02-22

**Authors:** Barbara Bodner-Adler, Engelbert Hanzal, Eleonore Pablik, Heinz Koelbl, Klaus Bodner

**Affiliations:** 1 Department of General Gynaecology and Gynaecologic Oncology, Medical University of Vienna, Vienna, Austria; 2 Section for Medical Statistics, Medical University of Vienna, Vienna, Austria; University of Insubria, ITALY

## Abstract

**Background:**

Vesicovaginal fistulas (VVF) are the most commonly acquired fistulas of the urinary tract, but we lack a standardized algorithm for their management. Surgery is the most commonly preferred approach to treat women with primary VVF following benign gynaecologic surgery.

**Objective:**

To carry out a systematic review and meta-analysis on the effectiveness of operative techniques or conservative treatment for patients with postsurgical VVF. Our secondary objective was to define the surgical time and determine the types of study designs.

**Methods:**

PubMed, Old Medline, Embase and Cochrane Central Register of Controlled Trials were used as data sources. This systematic review was modelled on the Preferred Reporting Items for Systematic Reviews and Meta-Analyses statement, including a registration number (CRD42012002097).

**Results:**

We reviewed 282 full text articles to identify 124 studies for inclusion. In all, 1379/1430 (96.4%) patients were treated surgically. Overall, the transvaginal approach was performed in the majority of patients (39%), followed by a transabdominal/transvesical route (36%), a laparoscopic/robotic approach (15%) and a combined transabdominal-transvaginal approach in 3% of cases. Success rate of conservative treatment was 92.86% (95%CI: 79.54–99.89), 97.98% in surgical cases (95% CI: 96.13–99.29) and 91.63% (95% CI: 87.68–97.03) in patients with prolonged catheter drainage followed by surgery. 79/124 studies (63.7%) provided information for the length of follow-up, but showed a poor reporting standard regarding prognosis. Complications were studied only selectively. Due to the inconsistency of these data it was impossible to analyse them collectively.

**Conclusions:**

Although the literature is imprecise and inconsistent, existing studies indicate that operation, mainly through a transvaginal approach, is the most commonly preferred treatment strategy in females with postsurgical VVF. Our data showed no clear odds-on favorite regarding disease management as well as surgical approach and current evidence on the surgical management of VVF does not allow any accurate estimation of success and complication rates. Standardisation of the terminology is required so that VVF can be managed with a proper surgical treatment algorithm based on characteristics of the fistula.

## Introduction

Vesicovaginal fistula (VVF) is an abnormal fistulous tract extending between the bladder and the vagina that allows the continuous involuntary discharge of urine into the vaginal vault. In addition to the medical sequelae from these fistulas, they affect physical, mental, social and sexual life of the patients [1]. In developing countries, the predominant cause of VVF is prolonged obstructed labour (97%) [1]. Conversely, in industrial countries iatrogenic injury to the urinary tract is the most common cause of VVF and the majority are consequences of benign gynaecological surgery [2]. It is estimated that 0.8 per 1000 of all hysterectomies are complicated by the development of a VVF [3]. Other causes in the developed world include malignant disease and pelvic irradiation [4]. In contrast to obstetric and irradiation fistulas, the typical postsurgical (post hysterectomy) fistula is the result of more direct and localised trauma to healthy tissue [5].

Although vesicovaginal fistulas (VVF) are the most commonly acquired fistulas of the urinary tract, we lack a standardized algorithm for their management [6]. Conservative management including prolonged bladder drainage, glue/fibrin injections, fulguration and so on is a reasonable option in cases with small, clean and non-malignant VVF [3,7]. Beside that, an operation is by far the most commonly preferred approach for affected women and the success rate varies between 75–95% with various different techniques in literature [3,8–13]. Multiple different surgical routes like Latzko repair, open transabdominal, transvaginal, laparoscopic, robotic, transurethral endoscopic with or without tissue interposition have been described [8,9,13], but no studies have compared surgical with conservative procedures and their outcomes in patients with VVFs following benign gynaecologic surgery. Furthermore, there is no general consensus regarding surgical time for a successful repair [7]. However, the evidence concerning treatment outcome with well-documented success and complication rates as well as the optimal surgical timing is lacking. To our knowledge, this is the first systematic review and meta-analysis investigating this topic. Primary outcome of interest was to review and summarize the current body of literature regarding effectiveness of disease management in patients with VVF following benign gynaecologic surgery. Our secondary objective was to define the most commonly reported time point for treatment and determine the types of study designs.

## Materials and methods

**Fig 1:**
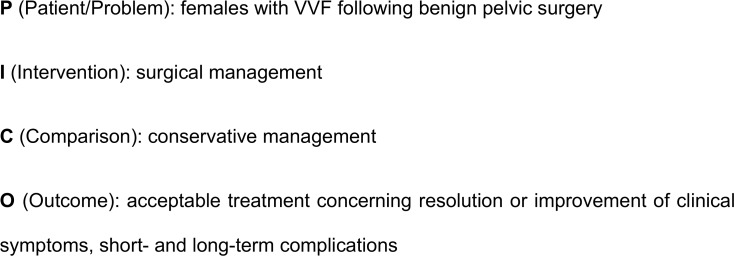
PICO question.

This study was reported following the Preferred Reporting Items for Systematic Reviews and Meta-Analyses (PRISMA) statement [14]. Before data extraction, the protocol of this review was registered with the PROSPERO International Prospective Register of Systematic Reviews (CRD42012002097) following the PRISMA guidelines for protocols (PRISM-P) [15]. The following PICO question was defined and is shown in Fig 1.

### Literature search

Literature search included 4 data sources using the retrieval systems DIMDI Classic search or OvidSp. In detail, we performed a computerised English-language Medline, Pub med, Cochrane Central Register of Controlled trials (CENTRAL) and Embase literature search using the MeSH terms *vesicovag* AND *fistul* AND (*management* OR *iatrogenic* OR *surgery* OR repair*), respectively. Our search ranged from 1947 to March 2016.

### Study selection

The limits for literature search were adult human females. Studies were included if they reported on a) vesicovaginal fistula b) which occurred after a benign gynaecologic surgery c) with clearly described conservative or surgical management. In screening process we excluded studies focusing on other types of urogenital fistulas (UGF), congenital fistulas or fistulas due to malignancy/irradiation or foreign bodies. Studies dealing with obstetrical VVF or trials, which did not clearly separate outcome parameters regarding fistula cause, were also excluded. Congress proceedings of international society meetings, textbooks, and review articles did not meet the inclusion criteria. Reports including men, neonates or adolescents despite the search limits were not included. Non-English articles with English abstracts were included if they provided information not found in English-language literature.

### Data extraction and study characteristics

Two investigators (BBA and KB) independently reviewed random titles and abstracts to establish reliable, reproducible inclusion criteria. All pertinent references from the manuscripts were obtained and reviewed. General characteristics were recorded from each study. Two authors (BBA and KB) independently abstracted study design, number of included patients, type or size of the VVF, different types of treatment (surgical/conservative), route and type of surgical treatment, cause of fistula and time point of surgical repair. The following outcome parameters were measured: time between fistula occurrence and repair (= surgical time), complete resolution of symptoms, success rate and treatment complications: postoperative leakage, de-novo stress incontinence, de novo urgency, urinary tract infection, number of attempts/repair, new-onset of pain/dyspareunia, recurrent VVF immediately (failure) or at any time postoperatively and long-term consequences on pelvic health including sexual function immediately or at any time after treatment. Terminology for success was inconsistent among included studies. We used terminology for success when success was either defined as “anatomical cure–fistula closed, healed or cured” or “absence of urinary loss, resolution of symptoms”. A total of 12 publications showed disagreement between the two reviewers. This was resolved by discussion with a third person (EH or HK). The findings of all relevant studies were abstracted, categorized and summarized by study design and outcomes measured. Furthermore, two of the authors (BBA and KB) independently rated the quality of the studies, using criteria from US Preventive Services Task Force and the NHS Centre for Reviews and Dissemination [16]. Studies received a poor rating if they were case reports, case series without adequate control group or comparative studies where the groups were not comparable.

#### Risk of bias (RoB) assessment

Risk of bias between included studies was independently assessed and evaluated by two of the authors (BBA and KB). Due to the types of study design of included studies the Newcastle Ottowa Scale for risk of bias assessment for comparative studies was used (Table 1) [17]. This considers 3 criteria (selection of study groups, comparability of groups and ascertainment of outcome of interest) for quality assessment. Discrepancy between the review authors over the risk of bias was resolved by discussion, with involvement of a third author where necessary.

**Table 1:** Quality assessment (Newcastle Ottowa Scale) for comparative studies.

Author, year	Selection	Comparability	Outcome/Exposure
Gupta N, 2010	***	*	***
Ou CS, 2004	***	**	***
Pshak T, 2013	**	*	***
Rajamaheswari N, 2012	***	**	***
Miklos JR, 2015	**	*	**

A study can be awarded a maximum of one star for each numbered item within the selection and outcome categories. A maximum of two stars can be given for comparability.

*: poor quality

**: moderate quality

***: high quality

### Synthesis of results

The meta-analysis was conducted on individual patient level using random-effect logistic regression models to calculate the probability of success for every type of therapy (conservative, surgical, combined) and every route and type of surgical treatment. 95% confidence intervals for the estimated proportion of successful treatments were calculated based on profile likelihood. To show the amount of heterogeneity the between trial variance τ² is presented for every model. Random- effects logistic regression models were used to manage study heterogeneity. Furthermore, calculation of the meta-analysis was also extended to random-effect logistic regression models. No odds ratios for the comparison between the different types of therapy were calculated as only 4 out of the 124 trials had a comparative study design while 120 studies reported uniform treatment for all documented patients. Therefore the differences in the outcome might be mainly influenced by the heterogeneity of the study populations. All statistical calculations were performed using the R-project for statistical computing (Version R-3.2.5) [18].

## Results

**Fig 2:**
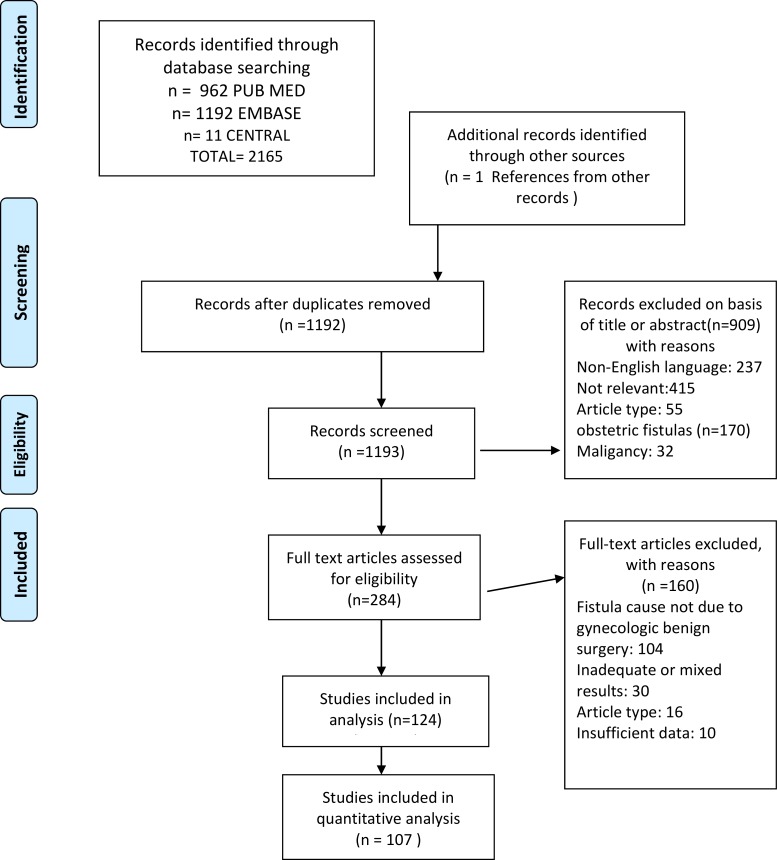
PRISMA Flow Chart.

We identified 2165 citations, reviewed 282 full text articles, and identified 124 studies for inclusion [1,4,8,10,13,19–137]. We excluded 1018 studies because they did not meet the inclusion criteria. The results of the search and screening procedure are presented as a PRISMA Flow Chart in Fig 2. The final analysis included 23 case reports, 95 retrospective case series, 5 comparative studies and 1 uncontrolled prospective study involving 1430 patients in all. There were no randomized controlled trials and no case-control studies. Case series contained between 2 and 110 patients. Detailed information of each included study (author, year, type of procedure and success rate) is summarized in Table 2.

**Table 2:** Included studies, type of procedure/approach and reported success rates.

Author, year	Type of procedure	sucess rate(%)
Ansquer et al, 200618	transvaginal	100%
Abdel-Karim et al, 201119	Laparoscopic	100%
Ayed et al, 200620	combined vaginal and suprapubic	41%
Aycinena et al, 197721	conservative (curretage)	100%
Agrawal et al, 201522	Robotic	100%
Blandy et al, 199123	Transabdominal	100%
Badenoch et al, 198724	Transabdominal	100%
Baumrucker et al, 197125	rubber ball	not stated
Bramhall et al., 195026	Transabdominal	not stated
Bajory et al, 201127	Transvaginal	100%
Brandt et al, 199828	Transvesical	96%
Bragayrac et al, 201429	Robotic	not stated
Clark et al, 197530	combined vaginal and transvesical	100%
Chibber et al, 200531	Laparoscopic	100%
Chien W-H et al, 200632	Transvaginal	100%
Chapron et al, 199533	Transabdominal	100%
Cruikshank et al, 198734	Transvaginal	82%
Chu Lei et al, 201535	Laparoscopic	100%
Dogra Prem et al, 201136	YAG laser weldging	88%
Dorsey et al, 196037	Transabdominal	100%
Daley et al, 200638	conservative (fibrin sealant)	100%
Dos Santos et al, 200839	Laparoscopic	not stated
Dorairajan et al, 200840	Transvaginal	not stated
Dalela et al, 200641	Transabdominal	100%
Ezzat et al, 2009 42	combined abdominal and vaginal	88%
Falk et al, 1957 43	conservative (electrocoagulation)	100%
Fourie et al, 198344	Transabdominal	88%
Flynn et al, 200445	Transvaginal	100%
Fearl et al, 196846	transvesical or transvaginal	90%
Fang et al, 201547	transvaginal (with foley catheter)	100%
Fleischmann et al, 198848	Transabdominal	100%
Gupta et al, 201049	transabdominal versus robotic	100%
Gözen et al, 200950	Laparoscopic	100%
Goodwin et al, 198051	Transvaginal	100%
Grange et al, 201452	combined vaginal and vesicoscopic	100%
Harrow et al, 196853	Transvesical	not stated
Hong HM et al, 201054	pointed electrocoagulation	100%
Hessami et al, 200755	Transadominal	100%
Hellenthal et al, 200756	Transabdominal	95%
Hemal et al, 200857	Robotic	100%
Henriksson et al, 198258	combined vaginal and suprapubic	78%
Hsieh CH et al, 200859	Transvaginal	1005%
Immergut et al, 195060	Transvesical	67%
Iselin et al, 199813	Transvaginal	100%
James et al, 201361	conservative (bladder drainage)	1005%
Javali et al, 201462	Laparoscopic	100%
Kostakopoulos et al, 199863	transvaginal and transabdomnal	100%
Krissi et al, 200164	fistulectomy & closure	not stated
Keettel et al, 197865	transvaginal and combined	94%
Kristensen et al, 66	Transabdominal	not stated
Ledniowska et al, 201267	transvaginal with modifications	not stated
Lazarou et al, 200668	Transvaginal	100%
Llueca et al, 201569	Laparoscopic	100%
Landes et al, 197970	Transvesical	100%
Dutto et al, 201371	Robotic	1005
Liao et al, 20124	Transvaginal	83,30%
Morgan et al, 195072	Transabdominal	not stated
DasMahapatra et al, 200773	Laparoscopic	100%
Modi et al, 200674	Laparoscopic	100%
Muto et al, 200575	conservative (glue)	66.6%
El-Lateef et al, 200376	Retropubic	100%
McKay et al, 200177	Cystorrhaphy	not stated
Milicic et al, 200178	Transvaginal	95.2%
McKay et al, 199779	Cystorrhaphy	100%
Miklos et al, 199980	Transvaginal	not stated
Malin et al, 196781	gold leaf	not stated
Moriel et al, 199382	Transvesical	100%
Mohseni et al, 201283	Transabdominal	86%
Macalpine et al, 194084	Transvesical	100%
Malmström et al, 195585	Conservative	100%
Mallikarjuna et al, 201586	laparoscopic (AINU)	100%
Miklos et al, 201587	Laparoscopic	97%(primary)100%(recurrent)	
Nagraj et al, 200788	Laparoscopic	not stated
Nabi et al, 200189	Laparoscopic	100%
Nesrallah et al, 199990	Transabdominal	100%
Nezhat et al, 199491	Laparoscopic	100%
Nerli et al, 201092	Transvesicoscopic	100%
Otsuko et al, 200893	Laparoscopic	not stated
Ou et al, 200410	combined /vag./abd./laparosc.)	83%/100%/100%	
Ostad et al, 199894	Transabdominal	100%
Phipps et al, 199695	Laparoscopic	100%
Persky et al, 197996	Transvesical	83%
Pietersma et al, 201497	Robotic	100%
Persky et al, 197398	Transabdominal	100%
Pontes et al, 197499	Transabdominal	100%
Peikoff et al, 1956100	Transabdominal	100%
Phsak et al, 2013101	Transvaginal	not stated
Rizvi et al, 2010102	Laparoscopic	100%
Reynolds et al, 2008103	Transabdominal	100%
Radopoulos et al, 2008104	Transvaginal	100%
Raz et al, 19938	Transvaginal	82%
Roslan et al, 2012105	LESS	100%
Razi et al, 2015106	combined(transvag./transabd.)	100%
Rader et al, 1975107	Transvaginal	100%
Rajamaheswari et al, 2012108	transvag. versus transabd.	95% vs.100%	
Szendi et al, 1959109	Transvaginal	100%
Schneidermann et al, 1958110	Suprapubic	100%
Shah et al, 2010111	conservative (fulguration)	not stated
Roen et al, 1960112	combined (transvag.,transvesic.)	not stated
Robles et al, 2009113	Transvaginal	not stated
Schimpf et al, 2007114	Robotic	100%
Sears et al, 2007115	Robotic	100%
Sundaram et al, 2006116	Robotic	100%
Starkman et al, 2007117	Transvaginal	100%
Singh RB et al, 2005118	Transabdominal	100%
Soong et al, 1997119	Transabdominal	67%
Sharma et al, 20141	Laparoscopic	100%
Singh V et al, 2013120	Laparoscopic	100%
Shirvan et al, 2013121	conservative (plasma/glue)	100%
Simforoosh et al, 2012122	Laparoscopic	75%
Sharifiaghdas et al, 2012123	Transabdominal	90%
Singh et al, 2012124	Transabdominal	89%
Tancer et al, 1992125	Transvaginal	89%
Toledo et al, 2013126	Observation	100%
Tsivian et al, 2006127	Transvaginal	100%
Tiong et al, 2007128	Laparoscopic	100%
Theobald et al, 1998129	Laparoscopic	100%
Taylor et al, 19481230	Transvesical	100%
Udea et al, 1977131	Suprapubic	100%
Wong et al, 2006132	Laparoscopic	100%
Wein et al, 1980133	Transabdominal	91%
Xu et al, 2005134	Transabdominal	100%
Zhang et al, 2013135	Laparoscopic	100%
Zumrutbas et al, 2014136	Cystoscopy	100%
Transvag.: transvaginal	transabd.: transabdominal	transvesic.: transvesical	

### Study characteristics

Fistula type was documented only in 58/124 (47%) studies. Of these, the majority of trials 35/58 (60%) dealed with simple fistulas, 21/58 (36%) with complex VVF and in a small percentage of studies (4%) complicated VVF were investigated. The majority of studies (66/124; 53%) did not comment on fistula type. Mean fistula size could not be calculated due to heterogeneity and insufficiency of data documentation. The majority of VVF occurred after a transabdominal hysterectomy (n = 943/1430; 66%), followed by vaginal hysterectomy (n = 126/1430; 9%), laparoscopic hysterectomy (n = 38/1430; 3%) and other benign gynaecologic operations (n = 72/1430; 5%). The remaining studies (17%) did not mention the type of hysterectomy causing the fistula. 46/124 (37%) studies included only patients who underwent a primary fistula repair (n = 221), 16/124 (13%) studies investigated patients who had previous attempts of fistula repair (n = 54) and 41/124 (33%) trials described a mixed collective of cases (n = 979). Remaining 21 studies (17%) did not give any information. Number of attempts varied between 1 and 3 repairs in average.

### Conservative treatment: Results of individual studies

10 studies described non-surgical treatment strategies as sole treatment option. These included transvaginal injection of fibrin sealant in 1 case, Yag Laser welding in 8 patients, cystoscopic electrocoagulation/fulguration/catheter method in 11 patients, endovaginal application of cyanoacrylic glue in 3 cases, platelet rich plasma/rich fibrin glue application in 6 women, curettage of fistula tract in 3 cases and ball technique with rubber/metal ball in 18 females. Success ranged between 67%-100% and the majority consisted of small VVF (<1 cm) [22,26,37,39,44,62,76,86,112,122].

239/1430 VVF (16%) were initially managed conservatively with prolonged catheter drainage (range: 2–12 weeks). Only 19/239 (8%) VVFs resolved with catheter drainage and the remaining 220/239 (92%) VVFs underwent surgical repair.

### Surgical treatment

The majority of patients were treated surgically. In all, 1379 patients were managed surgically and 97.98% (95%-CI: 96.13–99.29) were cured. The most commonly reported surgical approach was the transvaginal route (n = 534/1379; 39%), followed by a transabdominal/transvesical approach (n = 493/1379; 36%), a laparoscopic/robotic route (n = 207/1379; 15%) and a combined transabdominal-transvaginal approach in 45/1379 (3%) cases. Additionally, further various surgical techniques like transvaginal transurethral pointed electrocoagulation, transurethral suture cystorraphy, suprapubic cystotomy with gold leaf and so on were reported in 41/1379 (3%) cases. In 59/1379 (4%) VVFs the surgical route was not documented. Interposition grafts like Martius flap, Gracilis muscle, omental, peritoneal, labial fat flap or bladder mucosa autograft were used in the majority of studies (66 studies including 708 cases).

### Success after treatment

107/124 (86%) studies documented a success rate after treatment, describing 87 patients being completely symptom-free, 754 being completely dry and in 406 cases fistula healed completely or was cured.

#### Results of each meta-analysis with logistic regression model

Only studies which consistently evaluated treatment success were used for the meta-analysis. Success rate of conservative treatment was 92.86% (95%CI: 79.54–99.89), 97.98% in surgical cases (95% CI: 96.13–99.29) and 91.63% (95% CI: 87.68–97.03) in patients with prolonged catheter drainage followed by surgery. Success rates regarding surgical approaches were as follows: transabdominal/transvescial route 97.05% (95% CI: 94.55–99.18), transvaginal route 93.82% (95% CI: 89.96–97.49), laparoscopic/robotic approach 98.87% (95% CI: 96.85–99.99) and combined transabdominal/transvaginal route 90.70% (95% CI: 64.63–99.87). Use of interposition flap showed a success rate of 97.63% (95% CI: 95.31–99.22), without interposition flap reported success rate was 97.62% (95% CI: 93.63–99.91).

Reported frequency of success rates are summarised in Tables 3–8.

**Table 3:** Treatment modalities.

Treatment	conservativ	surgcial	Catheter and surgery
Number of studies	9 81,8% of 11	73 89,0% of 82	25 80,6% of 31
Number of patients	28 54,9% of 51	983 90,3% of 1088	239 82,1% of 291

**Table 4:** Frequency of success.

	Estimated proportion of successes %	95%-CI of proportion of successes	Between trial variance τ²
Conservative	92,86	79,54–99,89	0
Surgical	97,98	96,13–99,29	2,05
Catheter+surgery	91,63	87,68–97,03	0

**Table 5:** Surgcial approaches.

Surgical approach	Abdominal	transvaginal	Laparosc.or robotic		Others	combined	n.s.
Number of patients	493	534	207	41		45	59

**Table 6:** Frequency of success.

	Estimated proportion of successes %	95%-CI of proportion of successes	Between trial variance τ²
Abdominal/transvescial	97,05	94,55–99,18	0,49
Transvaginal	93,82	89,96–97,49	0,19
Laparoscopic/robotic	98,87	96,85–99,99	0
Others	100		
Combined	90,70	64,63–99,87	2,65

n.s: not stated

Laparoscp.: laparoscopic

**Table 7:** Use of interposition flap.

Interposition Flap	Without	with
Number of studies	21 72,41% of 29	62 93,94% of 66
Number of patients	217 64,98% of 334	695 98,16% of 708

**Table 8:** Frequency of success.

	Estimated proportion of successes %	95%-CI of proportion of successes	Between trial variance τ²
With Flap	97,63	95,31–99,22	1,594
Without Flap	97,62	93,63–99,91	2,034

### Intra- and postoperative surgical outcome

Successful intraoperative or postoperative outcome was mentioned in detail in 78/124 (63%) studies. The majority of these studies defined a successful outcome as an uneventful intra- or postoperative course and no immediate complications detected. 14 studies mentioned a complicated postoperative outcome and in 24 patients this was described in detail: ileus (n = 5), postoperative fever (n = 6), intraoperative bleeding (n = 2), grad II hydroureter (n = 1), clostridium difficile atelectasis (n = 1), wound infection (n = 2), bowel injuries (n = 2), compartment syndrome (n = 1), pelvic abscess (n = 1), and occurrence of ureterovaginal (n = 1) and vesicocolonic fistula (n = 2).

### Length of follow-up and complication rates

79/124 (64%) studies provided information for the length of follow-up. The remaining 45 studies did not mention any length of follow-up. The mean available follow-up time was 19.7 months. Complications were studied only selectively. Total number of studies mentioning complication outcome is shown in Table 9. Due to the inconsistency of these data it was impossible to analyse them collectively.

**Table 9:** Number of studies mentioning complication outcome.

Complications	Mentioned in Studies	Patients in these studies	Observed absolute frequency	Observed relative frequency
Failure/Recurrence	60	905	59	6,52%
UTI	15	229	12	5,24%
De novo SUI	26	247	5	2,02%
De novo pain	5	72	1	1,39%
De novo urgency	32	280	10	3,57%

### Long-term consequences and sexual function after treatment

None of the included studies documented any long-term consequences of pelvic health. Only 3 studies assessed sexual function after treatment [41,93,135]. Dorairajan et al. reported that 8/10 patients were sexual active without any signs of dyspareunia or pain [40], Nerli et al. reported that 2/4 cases were sexual active and all 3 women were sexual active in the study published by Xu et al. [93,135].

### Surgical time: Time between fistula occurrence and repair

In 22/124 (18%) studies, including 241 patients, surgery was initiated < 12 weeks after fistula occurrence. 15/124 (12%) studies with 223 patients defined the time point of surgical repair after 12 weeks. No statistically significant difference regarding success rate could be detected between early and late repair (p>0.05). 11 (9%) studies (n = 147 cases) started surgical timing < 12 weeks as well as > 12 weeks. The majority of studies (64/124; 52%), including 531 cases did not give any comment on their surgical time and 12 studies (9%) did not document an adequate time range.

## Discussion

Vesicovaginal fistulas are among the most distressing complications of obstetric and gynaecologic procedures, which can cause devasting medical, social, and psychogenic consequences [138]. The aetiology has changed, becoming more associated with hysterectomy. Despite numerous publications on this subject, the management of VVF remains a source of debate. The options of fundamental issues such as the preferred surgical approach and the optimal timing of surgery still vary widely [128].

### Main findings

This systematic review and meta-analysis assessed the effectiveness of disease management in patients with postsurgical fistulas and investigated treatment outcome with success and complication rates as well as surgical timing and type of study designs. The scientific literature consists mainly of case reports and retrospective case series. Furthermore, this analysis contains only a minority of studies, which used conservative treatment options as sole fistula treatment, as the majority of patients were treated surgically (96.4%). The preferred surgical approach was a transvaginal route, followed by transabdominal/transvesical approach, laparoscopic/robotic approach and combined operation techniques with reported success rates of 93.82%, 97.05%, 98.87% and 90.70%, respectively.

### Comparison with literature

Treatment of patients with VVF is currently controversial [139]. Although a trial of conservative management with prolonged bladder drainage might be tried, the spontaneous closure rate of VVF is low [51]. We found only 10 studies, which used conservative treatment as sole treatment strategy. Besides, our data confirmed that VVF resolves with prolonged catheter drainage only in a low percentage (8%). Some authors indicate that conservative treatment is only successful in smallest fistulas, and the majority of patients will require definitive surgical repair [3,44,140]. However, no studies exist comparing non surgical with surgical treatment strategies.

Although an operation is by far the most common recommendation for affected women, evidence concerning surgical treatment is lacking. Multiple different surgical techniques and approaches have been described in literature [8,9,13], but the choice mainly depends on location, severity and size of fistula [141]. Additionally, only few studies have compared the surgical procedures/approaches for VVF [10,50,102,109]. One study compared open and robotic surgical repair in patients with recurrent VVFs with no significant difference in outcome and complication rate [50]. Ou et al. evaluated three different surgical techniques (laparoscopic–open abdominal–transvaginal) in patients with primary fistula repair. Their data suggested that laparoscopic repair is feasible and results in lower morbidity than transabdominal and vaginal repair [10]. Phsak et al. compared the outcome between recurrent VVFs and primary VVFs without tissue interposition. The authors concluded that transvaginal repair of recurrent VVFs without tissue interposition is equally successful as in primary repairs [102]. Rajamaheswari et al. investigated the outcome between vaginal and transabdominal repair and reported comparable success rates between the two groups [109].

#### Surgical approach

The most important principle in repair is to provide tension-free, watertight closure, and the surgical route should be the one that provides the best possible chance of closure on the first attempt [142]. These principles can be achieved through a vaginal, abdominal or endoscopic approach. Although the choice of technique partly depends on the characteristics of the fistula, the surgical experience is also an important factor of successful outcome [138]. Although different surgical techniques have been described, a consensus for the ideal approach for surgical correction of VVF is still required [142].

#### Vaginal approach

In general, most gynaecologic surgeons prefer the vaginal approach, which has been associated with lower morbidity rates and with an equally good outcome [7,143]. The two most commonly reported vaginal repair techniques include Latzko technique and the layered closure [143]. This systematic review confirmed that vaginal fistula repair was used in the majority of cases with a reported success rate of 93.82%. Latzko operation was performed in 170 women and Tancer et al. published the largest investigation with 110 VVFs post hysterectomy. 107 patients were treated by partial colpocleises (Latzko repair) and 89% were cured at first attempt [126]. Although the included studies are inconsistent regarding characteristics of the fistula, we summarize that the vaginal approach for fistula repair is performed in the majority of female patients and therefore it is the surgical procedure with the highest level of experience in literature.

#### Abdominal approach

The abdominal route can be performed using a transvesical or an extravesical (bivalve technique) approach and is mainly indicated for loculated or complex fistulas [143]. We included 439 cases managed with an abdominal/transvesical approach with a reported success rate of 97.05%. Majority of these cases were treated with interposition graft. Due to the inconsistency of included trials and lack of fistula characteristics no recommendation can be made.

#### Laparoscopic and robotic-assisted approach

Minimally invasive laparoscopic surgery is increasingly being performed, including laparoscopic VVF repair [1,10,32,36]. In 2005, Chibber et al. described a laparoscopic approach to the O`Conor technique with reported advantages of decreased morbidity and a more rapid recovery [32]. One systematic review with 44 eligible studies compared the success rates between laparoscopic/robotic transvesical repair and extravesical laparoscopic repair techniques in patients with VVF. Due to their results, the authors summarized that extravesical VVF repair has similar cure rates compared to the traditional transvesical approach [144]. Most recent technology used in the treatment of VVF repair is the robotic-assisted approach and some authors reported excellent results with this operation technique [23,30,58,98]. Disadvantages include increased learning curve, time, costs and surgeons experience. We included 8 studies, which used a robotic-assisted approach. Success rates were excellent with 100% success, but number of included patients was small. Summarizing our data, due to the small number (n = 26 cases in all) and heterogeneity of studies, no clear statement and recommendation can be made regarding this operation technique and their success and complication rate in fistula repair.

#### Specified long-term outcome and complication rates

Postoperative complications are common and the most frequent postoperative complications reported in literature are de novo SUI, de novo urgency, leakage, de novo pain/dyspareunia, infection and failure [142,143]. Analysing our data, we could demonstrate that the majority of the included studies did not mention an adequate follow-up time and complications were described only selectively. We summarised 106/124 papers mentioning any complication, but from the remaining studies, which did not mention it we cannot assume that none occurred. Due to the inconsistency of these data it was impossible to analyse them collectively and no accurate prediction of complication rates can be made.

#### Surgical time

One of the main controversies in literature is the ideal timing for surgical intervention for postoperative VVF. Angioli et al. recommended waiting 2–4 months using continuous drainage of the bladder [143]. However, several other studies showed that, especially for small uninfected fistulae, early repair has better or at least similar success rates compared to delayed repair with additional advantage of reduced suffering and early commencement of normal life [24,25,143]. On the other hand, some reports indicate that timing of repair does not affect the outcome [145]. Our data demonstrated that 22 publications used an early repair, 15 studies started late surgical repair and 11 trials performed early as well as late repair. Due to this inconsistency, no serious recommendation can be done regarding ideal timing for surgical intervention.

#### Definition success rate and fistula classification

The reported cure rate of VVF varies between 75–95% with various different techniques in literature [3,8–13]. In accordance to literature, our findings revealed a success rate of 92.9% with conservative treatment, 97.98% in cases treated surgically and 91.63% in patients with prolonged catheter drainage followed by surgery. Summarising our data, no clear odds-on favorite regarding disease management as well as surgical approach could be identified and no technique was superior to any other. One major problem we faced was that success was defined in different ways, as many studies defined success as surgical closure of the fistula in place of function following surgery. In our opinion, successful surgical closure of the defect should be called ‘anatomical closure’ rather than ‘cure’, because many women suffer from on going pelvic organ, sexual and psychological dysfunction. Although this is of significant importance, only 3 studies [41,93,135] reported on sexual function after fistula treatment and the majority did not even mention this topic. Another problem that we faced when analysing the included studies was the lack of standardisation of terminology. The methodology of measuring the fistula as well as the used classification system was not clear in the majority of articles and none of the included studies stratified data by fistula type, primary repair versus previous attempts, fistula size or fistula location. Standardisation of the terminology is therefore required so that VVF can be properly managed [146]. Given the limitations of this analysis, future clinical research with a clearly defined VVF classification system, success definition better than anatomic result is needed to confirm our findings.

#### Quality and design of studies included

The scientific literature regarding surgical or conservative management of VVF following a benign gynaecologic surgery in female patients includes mainly case reports and retrospective case series and a variety of different surgical techniques. For this reason the majority of the included studies received a poor quality rating due to the study type. Furthermore, the reporting standard regarding surgical outcome, follow-up time and complication rate was poor. In addition to differences in reporting, an adequate documented follow-up time was not mentioned in the majority of cases, making it difficult to draw meaningful conclusions from these findings.

### Strengths and limitations

One of the strengths of our study is the inclusion of study data on the effectiveness of disease management in females with VVF in a specific population, namely after a benign gynaecologic surgery. The typical postsurgical fistula is the result of a direct and localised trauma to healthy tissue and therefore not comparable with obstetric or irradiation/cancer fistula. No similar analysis was found in literature. Besides, most of the included studies had the same primary outcome parameter, to be specific success after treatment. Limitations of our study are inherent to the limitations of the included studies. None of the included studies stratified data by fistula type, size or location. The methodology of measuring the fistula as well as the used classification system was not clear in the majority of articles. As no study reported data by using a unique classification system, a subgroup analysis according to fistula characteristics was not feasible. Another limitation arises from the study design as the majority of studies consisted of case reports or case series reporting uniform treatment for all documented patients. For this reason risk of bias assessment could be performed in a minority of studies with comparative study design. Furthermore, differences in outcome might be influenced by heterogeneity of study populations.

## Conclusion

Although the literature on disease management of females with postsurgical VVF is imprecise and inconsistent, our data show that the majority of patients are treated surgically through a transvaginal route. The quality and design of studies reviewed were weak with a poor reporting standard, weakening the conclusions that can be drawn. In summary, these data do not allow accurate prediction of success and complication rates in female patients with VVF following benign gynaecologic surgery. Standardisation of the terminology is required so that VVF can be managed with a proper surgical treatment algorithm based on characteristics of the fistula and well designed RCT are needed in future.

## Supporting information

S1 Checklist PRISMA 2009 checklist. (DOC)
